# Impact of the COVID-19 Pandemic on Early Intervention Services Use Among Children with Developmental Disabilities

**DOI:** 10.46889/jpar.2024.3101

**Published:** 2024-01-31

**Authors:** Jackson Kwok, Sherry Winston, Marsha Gerdes, Knashawn Morales, Ellen McQuaid, James P Guevara

**Affiliations:** 1 Department of Undergraduate Medical Education, Perelman School of Medicine, University of Pennsylvania, Philadelphia, PA, USA; 2 Division of General Pediatrics, Children’s Hospital of Philadelphia, Philadelphia, PA, USA; 3 Department of Biostatistics, Epidemiology and Informatics, Perelman School of Medicine, University of Pennsylvania, Philadelphia, PA, USA

**Keywords:** COVID-19, Children, Disabilities, Early Intervention

## Abstract

Early Intervention (EI) is a federally-funded program that provides therapies for children with developmental delays. Due to the COVID-19 pandemic and lockdown restrictions in Philadelphia in 2020, these services made a rapid change to virtual service delivery. We sought to explore the experiences of families in accessing online therapy. We recruited families to participate in a mixed methods study in order to evaluate changes in the initiation and use of EI services pre- and post-pandemic lockdown. Of the 94 who completed surveys, 27 families were purposively sampled for semi-structured telephonic interviews to identify their perceptions of program assistance and needs. Themes developed from these interviews included participant satisfaction with the communication of program changes and concerns related to the effectiveness of virtual EI services. This information can be used in plans for transitioning to hybrid therapy as well as for future pandemics requiring a return to fully virtual services.

## Introduction

Early Intervention (EI) is a service provided through Part C of the Individuals with Disabilities Education Act (IDEA) for children up to 3 years of age [1]. This federal program provides funding for physical, speech, occupational and other therapies with the goal of optimal child development and family competence and independence. The EI Part C Program is administered at the state or county level. In 2018, 3.5% of the 0 to 2- year-old population in the United States received EI services [2]. For Pennsylvania specifically, this number rises to 5.4% of the population [2]. On both the national and the state level, this percentage of infants and toddlers in EI has been increasing from previous years, though many children and especially those in urban environments still fail to receive services they are eligible for [3,4].

The COVID-19 pandemic and its associated mitigation strategies have resulted in numerous disruptions to the delivery of in-person healthcare services [5]. Visits to the office or other on-site locations were dramatically decreased with the onset of lockdown restrictions and this volume continues to remain lower than normal for many pediatric specialties [6]. Pandemic restrictions on in-person gatherings limited the day-to-day function of EI services, affecting not only at-home or on-site therapy visits but even the process of initiating care through referral or evaluation completion. In response to these changes, telehealth options offering provider visits through phone calls or video visits have seen a tremendous increase in usage [7]. The benefits of a socially-distanced online appointment during a pandemic are readily apparent and telehealth has become a staple tool in allowing patients to continue to safely seek necessary medical care [8]. For all its benefits, however, there are still ongoing hurdles to face in making telehealth a consistently viable alternative to in-person care. Some issues are inherent to the limitations of a virtual visit, such as the lack of available diagnostic equipment used in routine visits [7]. Others issues stem from problems with access; the all-virtual platform, for example, presents new technological and connectivity barriers for low-income families, potentially contributing to widening disparities in access to healthcare [9–11].

Several papers have already explored the processes and logistics behind the rapid virtual transition required following pandemic restrictions, as well as the benefits and challenges of increased telehealth utilization [12,13]. Our aims for this study were to determine changes in the receipt of EI services pre- and post-pandemic lockdown in the city of Philadelphia. In addition, we sought to explore the experiences and opinions of the program changes from the perspectives of low-income families using these modified services. This work has implications for how we manage the transition to post-pandemic EI with both virtual and some limited in-person services. This work also has implications for how to manage future pandemics necessitating a return to fully virtual visits: for both of these, participant perspectives will be invaluable for ensuring the effective delivery of these modified services.

## Material and Methods

### Participants

This study was approved by the Children’s Hospital of Philadelphia Institutional Review Board and the Philadelphia Department of Public Health Institutional Review Board. We recruited families from an ongoing randomized controlled trial testing the effectiveness of patient navigation among families of children less than 30 months old with suspected developmental delays and disabilities who were referred for EI services in Philadelphia from their primary care providers. Participants from this parent study were recruited from 6 primary care clinics in Philadelphia County. Participating families were consented by phone and completed an online survey [14]. The survey included questions concerning current and past year participation in Early Intervention (EI) services. We queried participants on program eligibility and use before and after the onset of pandemic lockdown restrictions in Philadelphia, March 16, 2020 [15].

To gain an in-depth understanding of program use and adjustment to virtual service delivery models, we purposively sampled participants for telephonic semi-structured interviews to identify their perceptions of program assistance and needs. Participants were queried regarding current participation, how services were delivered (e.g. virtually or in-person), whether the current format met their needs, about barriers and facilitators to program participation and what unmet needs they currently had. We developed and pilot tested an interview guide prior to the first interview. All interviews were audiotaped and transcribed for further analysis. Qualitative analysis of the transcripts was based on inductive coding using a consensus approach. Three authors (JK, SW, JPG) reviewed all transcripts to develop consensus on codes. Once consensus on codes was reached, all transcripts were recoded and a 25% sample of transcripts was selected to assess agreement, which yielded a >70% intercoder agreement. Themes were identified from the codes using modified Grounded Theory.[16] We continued the interviews until thematic saturation, i.e. no emergence of new ideas. We used NVivo, a qualitative software program, to code all transcripts and complete qualitative analysis.

### Outcomes

Our main outcomes were referral completion and service initiation. In our study, families who completed referrals prior to March 3, 2020 were included in the pre-pandemic category regardless of when services were initiated. Those families with referrals initiated after March 3, 2020 were included in the post-pandemic category. Those participants with both a pre-pandemic completed referral and a second referral initiated later were listed in both categories. Analysis. Survey results were exported to Stata Statistical Software, version 15, for cleaning and analysis. We used the Chi-square statistic to evaluate differences in use of EI services during the pre-pandemic and post-pandemic time periods. P-values less than 0.05 were considered statistically significant. We developed a logistic regression model to assess differences in EI referral completion and service initiation by time period while controlling for participants’ age, race/ethnicity, family income and their allocated intervention arm in the parent study.

## Results

We contacted 128 families from the parent study who were eligible for recruitment. Of these, 98 participants were enrolled in the study and 94 of these enrolled families completed the online survey. From among those that completed the survey, 27 participants were purposively sampled and selected for qualitative interviews. Participants in the study were predominantly women (93%), self-identified as Black (71%) and had family incomes below $55,000 (78%) (Table 1). These characteristics were similar to the overall demographic characteristics of participants in the parent study. Each arm of the parent study was equally well-represented among participants, with 49% of surveys coming from the control arm and 51% of surveys from the intervention arm, in which participants received additional support to navigate the EI system (Table 1). Survey results showed that 55 participants (63%) reported they had initiated referrals for EI services. Of these, 46 (84%) reported they had completed their EI referral and started services. Fifty-two families (62%) reported they had initiated referrals in the pre-pandemic period, but only 15 families (18%) initiated referrals in the post-pandemic period and 12 (80%) applied in both time periods (Table 2). Referral completion rates were similar in the pre-pandemic and post-pandemic period: 79% (41 families) and 80% (12 families), respectively (Table 2). After adjusting for participant age, race, income and study arm, we found that EI service usage was less likely in the post-pandemic period (Adjusted OR 0.16, 95% CI 0.03 – 0.99) (Table 3). We also found that EI service usage was more likely for those participants in the intervention arm (Adjusted OR 6.61, 95% CI 1.04 – 42.20). Qualitative analysis of interviews identified 6 primary themes: (1) Successful communication of changes; (2) Successfully answering parent concerns; (3) Satisfaction with the length and pace of virtual visits; (4) Perceived decrease in the effectiveness of virtual therapy; (5) Logistical hurdles unique to online sessions; (6) Preference for in-person services with the potential for a hybrid model of delivery. Each theme is described below.

1. EI was successful in communicating pandemic-related program changes to its participants. Communication between the EI program and participating families was a strong point of the pandemic transition. Parents overwhelmingly reported that they were provided with frequent program updates and given the opportunity to switch to virtual visits with little disruption in services. Respondents noted that EI used multiple modes of communication to convey program information and program changes, including phone calls, texts and mail. Service Coordinators for EI were also cited as a frequent and reliable source for updates (Table 4). Day-to-day changes in services were often managed directly between parents and providers through phone calls or texts, which respondents noted as another good aspect of the EI program’s communication strategy during the pandemic. Maintaining these direct channels between parents and service providers was perceived to be useful in managing last-minute schedule changes or navigating further updates as virtual sessions were initially being established. This direct line of communication was perceived as allowing for increased flexibility in how services were provided, aided by the at-home nature of online visits (Table 4)
2. EI was successful in answering parent queries and addressing parent concerns. Parents often talked about their experiences asking questions to their service coordinators and providers, usually through phone calls or texts. Respondents generally felt that when they had questions or concerns about EI, program staff were responsive. These perceptions were expressed by both long-time users of EI services and those who were relatively new to the program. This ease of access for assistance was often noted by parents to be in contrast to experiences with other programs, resulting in a high level of satisfaction with the quality of help received (Table 4)
3. Families were satisfied with the length and pace of virtual visits and appreciated the ability to see the provider’s face. Parents were initially concerned that the loss of home or childcare visits and subsequent transition to online appointments would lead to fewer and shorter visits as the EI program adjusted to these new lockdown hurdles. However, parents noted that their appointment frequency did not experience any severe or long-term disruptions. Concerning the duration of these virtual visits, parents perceived that the time their child spent with therapists and providers was comparable to their previous in-person experiences and was appropriately paced (Table 4). For some respondents, their overall satisfaction with appointment duration and pacing was noted in the context of their child’s attention span which, as we note in the next section, was perceived by many parents to be relatively short during virtual sessions (Table 4). One positive aspect of virtual visits that parents perceived was the ability to see the provider’s face on the screen, as opposed to having faces obscured by masks in other types of in-person interactions during the pandemic lockdown restrictions (Table 4). For these parents, the ability to remove masks was important in increasing child comfort and reducing stranger anxiety during sessions, especially with therapy focused on social interactions
4. Parents perceived that virtual therapy was less effective due to increased distractions and lack of engagement. One consistent issue that parents raised with the use of virtual visits was the perception that it was more difficult for their child to maintain focus during sessions conducted over the computer (Table 4). The lack of an in-person provider to maintain their child’s attention during sessions was perceived as a major detriment of the virtual platform. Parents felt that it became their responsibility to maintain their child’s attention during telehealth appointments, which has been another source of frustration. (Table 4). For those families that had EI services prior to the pandemic lockdown, many felt that their child was less distractible and more focused with in-person providers. The switch to virtual, parent-led sessions led to a perceived decrease in child engagement during sessions. Many parents expressed concerns that a decrease in engagement lead to less effective therapy overall when conducted online, although they did acknowledge the need for these virtual sessions during the pandemic lockdown. Parents noted that their child did continue to show improvements with virtual services, especially those children that had already established a therapeutic relationship with their provider through previous in-person sessions. Even in this context, however, parents felt that their child’s rate of improvement was slower with online learning versus home or childcare visits (Table 4)
5. There were logistical hurdles to online sessions, including connectivity issues and lack of equipment. Connectivity issues were frequently cited as a source of frustrations unique to the virtual space. Some parents were limited to using their phones for appointments due to a lack of computer or tablet access; the smaller screen, in turn, made it harder for parents to keep their child’s attention on the provider, contributing to a perceived drop in engagement (Table 4). Internet access and stability were mentioned as barriers to successful virtual visits, with many appointments being cut short due to inconsistent connections (Table 4). Lack of therapeutic equipment was another common issue raised by parents with regard to virtual therapy. When sessions were in-person, providers used specialized tools directly with the child during their appointment, which parents perceived as being a key aspect in maintaining attention and in facilitating improvement. Some interim workarounds were developed: providers used screen sharing to show these toys or books virtually and in some cases, parents purchased the equipment themselves. But for low-income parents, the added expense of purchasing such equipment was simply not an option, leading to parent perceptions of an inferior session experience for their children (Table 4)
6. Parents had a preference to return to in-person services but were open to a hybrid model of delivery if a child-provider relationship was well-established. When asked about their preferences for future delivery of EI services, most parents expressed a desire to return to in-person therapy sessions with their provider once lockdown restrictions ended. A major reason for parents wanting more home or daycare visits was the increased social interaction and engagement associated with in-person appointments. A second reason was parents’ hesitation and reluctance to carry out the therapies themselves. (Table 4). Though interest was low for an entirely virtual EI, there was interest in a hybrid model of services that combined both online and in-person components. For such a hybrid model, respondents felt that different provider-led exercises and hands-on activities would be better suited for home or daycare visits, but other types of therapy could be completed online (Table 4). Parents felt that a hybrid model would only be a consideration after a child-provider relationship had been well established (Table 4). Meeting their therapist beforehand and having that bond, parents reasoned, would be important to keeping engagement up even when there was only a screen to interact with during virtual sessions.

## Discussion

The in-person provision of EI services in Philadelphia was greatly impacted by lockdown restrictions associated with the COVID-19 Pandemic, with a rapid transition from in-person to a model of fully virtual appointments. Our survey found that participants sought EI services more frequently in the pre-pandemic period compared to the post-pandemic period but similarly received services during both time periods. The ability to maintain similar referral completion rates following lockdown restrictions indicates that the use of virtual platforms for the intake process was successful. This may prove to be beneficial for a broader utilization of early intervention, allowing those in rural areas or who otherwise cannot have in-person evaluations the opportunity to still be evaluated for services.

Research on the process of establishing a telehealth system has covered the importance of smooth service implementation in order to improve overall adoption, typically from an internal health systems perspective [17–19]. Our study explored the public-facing side of establishing a virtual services platform, with firsthand experiences on where programs may have succeeded or failed in making the transition. One consistent finding from these interviews was the importance of transparency. Parents often cited Early Intervention as a program that prioritized communicating their plans for virtual therapies, which both made it easier to implement the transition and also allowed for parents to access assistance with the change in advance of their appointments. This was especially apparent to those that struggled to obtain a similar level of engagement with other public programs. These themes underscore the role of proper program communication and offering multiple avenues for assistance, two strengths of EI implementation that had a positive impact on participants’ experiences with the transition to virtual services.

Several studies have explored perceptions around virtual teaching environments and their propensity for interruptions, especially for child learners [20–22]. Comments expressing dissatisfaction with telehealth services cited this difficulty with consistent engagement as a key drawback to the service. However, Philadelphia Infant-Toddler Early Intervention has established goals of training parents to be teachers with providers acting less as individual therapists and more as interventionists teaching families how to help their child [23,24]. In such model, full attention and full engagement with an online platform is not necessary or expected. Our responses show that parents often do not share this perspective and were still trying to maintain the same level of individual therapy whether in-person or virtually. Parental opinions and the environmental support they provide are also vital to EI services; not just so that they continue to participate, but because their perceptions and expectations may directly impact therapy participation [25].

In the future, providers may benefit from better training in describing the intended EI model and in setting expectations for virtual therapy sessions. Increased utilization of EI services is an important factor in improved outcomes for children with developmental delays [26].

Disruptions in the process of obtaining EI services are a common source of parent frustration and can contribute to decreased usage; in some cases, these hurdles can prevent the initiation of services entirely [27,28]. With the introduction of online-only therapy appointments, there were several issues unique to the virtual platform. Some of these problems were centered on the physical equipment required for telemedicine; this included both equipment needed to host video calls, as well as therapeutic tools used by providers during typical in-person sessions. Availability of therapeutic equipment further exacerbated parent concerns regarding child participation, as they perceive these tools to be important interactive components of a therapy session. Options to solve both of these problems are often limited to purchasing the required components. At the time of these program changes, however, many families faced increased rates of unemployment and other sources of financial strain [29]. Usage of funds for devices caple of effective connectivity may be an option in the future. Additionally, the provision of specialized equipment for therapy to parents may be a method of increasing parental satisfaction and buy-in towards their role as teacher, which would help to mitigate concerns of engagement and potentially increase the perceived effectiveness of their online sessions.

Studies published after the start of the COVID-19 pandemic have examined telehealth and online delivery of medical services following the gradual easing of lockdown restrictions [30,31]. Options in this regard range from keeping all services completely virtual to returning to in-person appointments. For many participants in our study, the preferred choice was a hybrid model utilizing online visits when feasible while still having some in-person components as necessary. One feature of such a hybrid model would be in-person visits for the first few therapy appointments followed by virtual visits. Based on their experiences, parents felt that their children were more likely to engage with providers, even in a virtual environment, when the child had developed that relationship and was generally more excited to see their therapist. Our work found that parents were open to and even interested in a hybrid model that combines in-person elements to increase engagement with online elements that allow for more flexibility in scheduling.

Our study had several limitations. First, our sample population was drawn from a larger parent study for which the intervention arm was provided with patient navigation for completion of EI referrals. Second, we had a relatively small sample size for the quantitative analysis. Third, this study also lacked generalizability as our research was restricted to a a single urban county. Our study, however, had important strengths. First, we conducted first-person interviews with participants about the barriers and facilitators that they experienced in trying to obtain and maintain services during lockdown restrictions. Second, we identified areas where EI was thought to be successful and where parents perceived a decline in quality. Maintaining positive aspects of the transition while targeting these deficits will be an important area of focus as we begin to see the return of some in-person services and also as we prepare for future pandemics or other scenarios where virtual-only services are once again necessitated.

## Conclusion

The delivery of EI services in Philadelphia and nationwide was drastically changed in the wake of lockdown restrictions. We found that parents who sought EI referrals after the pandemic started were less likely to initiate referrals compared to those who sought pre-pandemic. For those already enrolled in EI, the program had to make sweeping changes to its practices in a relatively short timeframe. Our findings show that families felt as though they were supported and adequately communicated with by EI during this transition period. Service coordinators and direct lines of communication with providers were vital to maintain this flow of information and updates as the situation developed. Barriers in virtual therapy that did arise included a perceived drop in child engagement as well as issues with a lack of equipment that was previously used for in-person appointments. Work should be done to address concerns of decreased engagement and child participation with virtual therapy. Moving forward, families expressed a willingness to adopt a hybrid model of EI service delivery, with both in-person and telehealth components. Information from this study offers insights on how to improve the online aspect of EI therapy, both for use in such a hybrid model and also for future pandemics that may require a return to completely virtual services.

## Figures and Tables

**Table 1:** Demographic data for EI survey respondents.

Characteristic	Number of Respondents (%)
**Age, N=84**	
< 29 years	24 (29%)
29–38 years	49 (58%)
> 39 years	11 (13%)
**Sex, N=84**	
Female	78 (93%)
Male	6 (7%)
**Race, N=84**	
Black	60 (71%)
White	14 (17%)
Hispanic	3 (4%)
Other	7 (8%)
**Yearly Income, N=84**	
Less Than $25,000	39 (46%)
$25,000 to $55,000	27 (32%)
Greater than $55,000	18 (22%)
**Arm of Parent Study, N=84**	
Control Arm	41 (49%)
Intervention Arm	43 (51%)

**Table 2:** REDCap survey responses for pre- and post-pandemic application and usage of EI.

Overall	Number of Respondents (%), N=84	
	Total Pre- and Post-Pandemic	
**Applied for Service** (% of Total N)	55 (65%)	
**Received Service** (% of Applied)	46 (84%)	
	Pre-Pandemic	Post-Pandemic
**Applied for Service** (% of Total N)	52 (62%)	15 (18%)
**Received Service** (% of Applied)	41 (79%)	12 (80%)

**Table 3:** Logistic regression analysis of factors in EI service initiation*.

	OR	SE	Z	P > |Z|	95% CI
**Time Period**					
Pre-Pandemic	REF	REF	REF	REF	REF
Post-Pandemic	0.16	0.15	−1.98	0.048	0.03, 0.99
**Arm of Parent Study**					
Control	REF	REF	REF	REF	REF
Intervention	6.61	6.26	2.00	0.046	1.04, 42.20
**Participant Age**					
< 29 y/o	REF	REF	REF	REF	REF
29–38 y/o	0.86	0.90	−0.14	0.886	0.11, 6.74
>39 y/o	1.11	1.58	0.07	0.941	0.68, 18.10
**Participant Race**					
Black	REF	REF	REF	REF	REF
White/Hispanic/Other	2.59	2.80	0.88	0.381	0.31, 21.60
**Participant Income**					
< $25,000/yr	REF	REF	REF	REF	REF
$25,000–55,000/yr	0.61	0.57	−0.53	0.598	0.01, 3.83
> $55,000/yr	0.39	0.50	−0.73	0.462	0.03, 4.78

* Results of the logistic regression model of factors with relation to EI referral completion and service initiation. OR: Odds Ratio; SE: Standard Error; CI: Confidence Interval; Ref: Reference Level.

**Table 4:** Thematic analysis of semi-structured interviews.

Theme	Representative Quotes
**EI has been successful in communicating pandemic-related program changes to its participants.**	“The service coordinator was good… she started talking to us about it (lockdown restrictions) like two weeks before. Like, hey, this shutdown may happen”. “If he (her son) just decides that he doesn’t want to wake up at 9 o’clock today, he wants to wake up at 11:00, I could always just text her or send her an email or call her (the provider) and say well, can we start a little later than nine and she could say okay”.
**EI was successful in answering parent questions and addressing parent concerns.**	“Early Intervention is better. It’s the best of all we’re talking about now because it’s easy. You can reach everybody on the phone whenever you want”. “And whenever I voiced my concerns… they’ll get me in touch with somebody higher than who I’ve been dealing with. They’ve really been trying to work with me”.
**Families were satisfied with the length and pace of virtual visits when compared to in-person visits and liked that their children could see the provider’s face.**	“We get 45-minute sessions once a week and they’re never rushed. Like sometimes they go a few minutes beyond.” For some respondents, their overall. “The length of sessions is fine for us. It’s like 45 minutes and that’s fine… my child is 18, 20 months, anything longer than that would not be doable”. “So actually, doing this virtually has been very nice, because we’re able to see the therapist but without having them physically present in the home and isn’t wearing a mask”.
**Parents felt like virtual therapy was less effective due to increased distractions which led to slower rates of improvement in their child.**	“A 3-year-old is not gonna sit there and talk to the person – they’ll probably run around or something or be playing with other things. Their attention is not gonna be on the computer”. “It’s hard for me to keep him focused or to try to get him to interact with the stuff that the teacher would want him to interact with on the computer”. “He came a long way on that program when he was meeting with his therapist in person, so I didn’t want to cut all the way… and he made some strides with the online, but not as great as before the pandemic”.
**There were logistical hurdles unique to online sessions, including connectivity issues and lack of equipment typically used in therapy sessions.**	“If I was able to maybe get like a computer, if the therapist was on like a bigger screen, he would be more focused into looking at her as opposed to it being on my phone”. “If the internet connection kind of moves the whole session is kind of just done with because now, we can’t hear them. They can’t hear us”. “If a parent can’t afford something and to have for that child to communicate with the therapist over the computer, then the child is not gonna be as entertained to interact with the therapist”.
**Parents generally would like to return to in-person services, but may be open to a hybrid model of delivery if a child-provider relationship is already well-established.**	“She (the therapist) sends little assignments and I print them out and I do it with him, but it was just a lot better when he was being helped from the actual both of us instead of just me”. “I do think that the social engagement, especially with speech therapy – I would probably go back to the in-person visit, it’s really hard to get to know a 2-year-old over the computer”. “I would do virtual most of the time. The only time I would do in person is for the different exercises different things that I feel as though my child needs to be hands-on for”. “I guess we could meet (the therapist) a couple of times and so my son can get to know her and then we can try a couple of online sessions and see how it goes”.
